# Physical Frailty as a Barrier to Improvements in Physical Performance in Older Patients With Heart Failure Undergoing Acute-Phase Cardiac Rehabilitation

**DOI:** 10.7759/cureus.88846

**Published:** 2025-07-27

**Authors:** Yuji Hongo, Kazufumi Kitagaki, Rie Futai, Takeshi Hasegawa, Hiroshi Morikawa, Hisashi Shimoyama

**Affiliations:** 1 Department of Medical Technology, Itami City Hospital, Hyogo, JPN; 2 Faculty of Rehabilitation, Shijonawate Gakuen University, Osaka, JPN; 3 Department of Cardiovascular Medicine, Itami City Hospital, Hyogo, JPN

**Keywords:** cardiac rehabilitation, frailty, heart failure, older patients, physical performance

## Abstract

Introduction

Poor physical performance is associated with an increased risk of post-discharge cardiovascular events in patients with heart failure. In this study, we investigated the association between physical frailty and improvements in physical performance through cardiac rehabilitation in hospitalized older patients with heart failure.

Methods

The study included 100 patients with heart failure (aged ≥65 years) hospitalized between January 2023 and August 2024, with a short physical performance battery (SPPB) score ranging from 1 to 11 points at the initiation of cardiac rehabilitation. Patients achieving an improvement of ≥1 SPPB points during hospitalization were classified as the improved group, while those with unchanged or declining scores comprised the non-improved group. We retrospectively examined the association between physical frailty and improvement in physical performance due to acute-phase cardiac rehabilitation during hospitalization.

Results

Among the 100 patients, 62 and 38 were categorized into the improved and non-improved groups, respectively. Although no significant differences were observed regarding age, sex, or rehabilitation duration between the groups, the prevalence of physical frailty was significantly higher in the non-improved than in the improved group. Modified Poisson regression analysis, controlling for age, sex, and New York Heart Association functional class, showed that physical frailty was significantly associated with a reduced likelihood of improvement in physical performance.

Conclusion

Physical frailty may inhibit improvement in physical performance among hospitalized older patients with heart failure. Therefore, enhancing acute-phase rehabilitation strategies for patients with frailty and strengthening the post-discharge follow-up system are essential.

## Introduction

The global number of patients with heart failure is estimated to be approximately 64 million, and this number is expected to increase further as the population continues to age [1]. The average age of hospitalized patients with heart failure has increased from 70.7 to 78.0 years between 2004 and 2013, indicating a trend toward older patients with heart failure [2,3].

Older patients with heart failure often have multiple comorbidities, such as diabetes, hypertension, or chronic kidney disease, which increase the risk of developing physical frailty through mechanisms like systemic inflammation. This highlights the urgent need for effective strategies [4,5]. Additionally, patients with physical frailty are more likely to experience impaired physical performance, an independent risk factor for cardiovascular events [6]. Therefore, maintaining optimal physical performance in older patients with heart failure is essential.

Guidelines recommend cardiac rehabilitation in patients with heart failure as it effectively enhances exercise tolerance and physical performance in these patients [7]. The REHAB-HF trial (A Trial of Rehabilitation Therapy in Older Acute Heart Failure Patients) found that a 12-week post-discharge rehabilitation program improved physical performance in older patients with heart failure, while similar benefits were observed for patients with frailty [8]. Thus, the advantages of outpatient cardiac rehabilitation for patients with and without frailty are becoming increasingly well-established.

Despite previous research, the effectiveness of acute-phase cardiac rehabilitation for patients with physical frailty has not yet been adequately explored [9]. Approximately 45-56% of hospitalized older patients with heart failure are complicated by physical frailty [4,10], indicating the importance of examining the effectiveness of cardiac rehabilitation in patients with frailty. Since acute-phase cardiac rehabilitation has been shown to reduce hospital stay duration and improve activities of daily living [11], our institution initiates cardiac rehabilitation as early as possible after admission. In this study, we aimed to retrospectively investigate the association between improvements in physical performance and frailty resulting from inpatient cardiac rehabilitation.

## Materials and methods

Study design and participants

**Figure 1:**
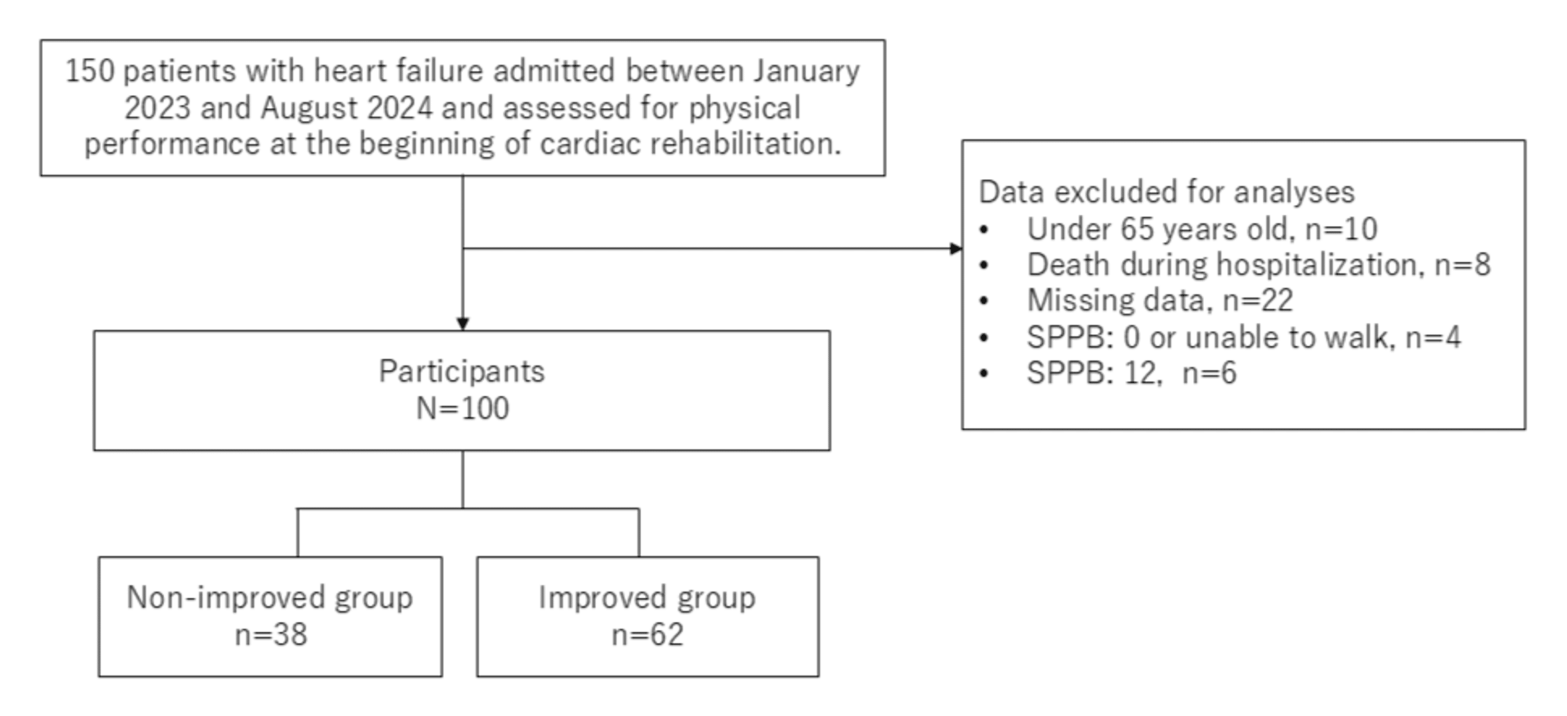
Flow diagram of the patient selection process in this study The flowchart describes the patient selection process for inclusion in the study, beginning with 150 hospitalized patients with heart failure. Patients who were aged <65 years, had missing data, died during hospitalization, or scored 0 or 12 on the short physical performance battery (SPPB) were excluded, leaving 100 participants eligible for analysis.

Figure 1 presents the study participant selection flowchart for this single-center retrospective observational study. In total, 150 patients with heart failure were admitted to the Cardiology Department of Itami City Hospital between January 2023 and August 2024 and assessed using the short physical performance battery (SPPB) at the initiation of cardiac rehabilitation. The exclusion criteria were: an age less than 65 years, death during hospitalization, missing data, being unable to walk or having an SPPB score of zero, and an SPPB score of 12 (full score). Patients with an SPPB score of 12 were excluded from the analysis to avoid ceiling effects, as these individuals already demonstrated optimal physical performance at baseline. After applying the exclusion criteria, 100 patients remained for analysis.

The patients were enrolled in cardiac rehabilitation as soon as possible after admission. The rehabilitation program was implemented in accordance with the Guidelines for Rehabilitation in Cardiovascular Disease [7], beginning with early mobilization while carefully monitoring hemodynamic stability to prevent the adverse effects of prolonged bed rest. As the patients’ symptoms and physical condition improved, the program progressed to include low-intensity aerobic exercise, resistance training (targeting a Borg scale of ≤13), as well as training in activities of daily living and balance, tailored to individual functional status. In accordance with previous studies, aerobic exercise was classified as training conducted with specific exercise equipment only [12]. The SPPB was measured by a physical therapist at the initiation of cardiac rehabilitation and again at discharge. Based on the minimum clinically meaningful change of one point in the SPPB [13], patients whose SPPB score improved by at least one point at discharge when compared with the initial measurement were categorized as the improved group, while those whose SPPB remained unchanged or decreased were classified as the non-improved group.

Measures of physical performance and frailty

Physical performance was assessed using the SPPB, which was measured early in the cardiac rehabilitation process (within three days of being cleared to walk by the attending physician) and again within the last three days before discharge.

The SPPB is an index of physical performance that consists of three items: balance, gait, and stand-up tests [14]. Each item is rated on a five-point scale from zero to four, with a higher total score indicating better performance. The balance test was scored according to the holding time in the closed-leg standing, semi-tandem standing, and tandem standing positions, while the gait test was scored according to the shortest value of the normal walking time in a four-meter section without assistance, measured in duplicate. The stand-up test was scored according to the time taken to stand up from a 40-cm-high chair, with the arms crossed in front of the chest, five times.

Physical frailty was assessed based on the revised Japanese version of the Cardiovascular Health Study criteria (J-CHS), which included weight loss (≥2 kg in six months), low grip strength (<28 kg for men, <18 kg for women), fatigue, slow gait (<1.0 m/sec), and inactivity (exercise frequency less than one time per week) [15]. Patients who met at least three of these criteria were classified as having physical frailty. In contrast, social frailty was defined as the presence of two or more items in Makizako's criteria [16], while cognitive frailty was defined as a Mini-Cog score of less than three [17,18].

Statistical analysis

All analyses were performed using R software (version 4.1.1; R Foundation for Statistical Computing, Vienna, Austria), with statistical significance set at a P-value of <0.05. Categorical variables, presented as frequencies and percentages, were analyzed using the χ2 test or Fisher's exact test, while continuous and ordinal variables, presented as medians and interquartile ranges (IQRs), were compared using the Mann-Whitney U test.

Multivariate analysis using modified Poisson regression was conducted to calculate risk ratios (RRs) and 95% confidence intervals (CIs) [19], with the outcome and independent variables being the change in the SPPB score and physical frailty, respectively. Age, sex, and the New York Heart Association (NYHA) classification were included as confounders [20,21].

## Results

Patient characteristics

Among the 150 patients who met the inclusion criteria, 100 were included for analysis after applying the exclusion criteria. Table 1 presents the patient background characteristics. Of the 100 patients (median age: 83 years (IQR: 77-89)), 45 (45%) were male and 62 (62%) showed improved physical performance in the SPPB at discharge when compared with that at the initiation of cardiac rehabilitation.

**Table 1:** Clinical characteristics of the non-improved and improved groups Data are expressed as the median (interquartile range) and No. (%). Categorical variables were analyzed using the χ² test or Fisher’s exact test, as appropriate. Continuous and ordinal variables were analyzed using the Mann–Whitney U test. Alb; albumin; BMI, body mass index; BNP, brain natriuretic peptide; eGFR, estimated glomerular filtration rate; Hb, hemoglobin; HFpEF, heart failure with preserved ejection fraction; IHD, ischemic heart disease; LVEF, left ventricular ejection fraction; NYHA, New York Heart Association; VHD, valvular heart disease

	All (N=100)	Non-improved group (n=38)	Improved group (n=62)	Statistic (test)	P-value
Age, years	83 (77–89)	86 (78–91)	82 (77–88)	U=864	0.03
Sex, male	45 (45%)	13 (34%)	32 (47%)	χ²=2.2	0.14
BMI, kg/m^2^	23 (20–25)	23 (19–25)	23 (21–26)	U=974	0.42
Etiology					
VHD	27 (27%)	10 (26%)	17 (27%)	χ²=0	1.00
IHD	31 (31%)	7 (18%)	24 (39%)	χ²=3.6	0.06
Arrhythmia	60 (60%)	23 (61%)	37 (60%)	χ²=0	1.00
Cardiomyopathy	14 (14%)	6 (16%)	8 (13%)	χ²=0.01	0.92
Others	6 (6%)	4 (11%)	2 (3%)	N/A	0.20
BNP, pg/mL	473 (265–938)	463 (278–685)	537 (247–969)	U=1053	0.78
Alb, g/dL	3.5 (3.1–3.7)	3.4 (3.0–3.7)	3.5 (3.3–3.7)	U=895	0.11
Hb, g/dL	12 (10–13)	10 (10–12)	12 (11–13)	U=772	0.003
eGFR, %	43 (31–61)	39 (26–60)	48 (35–61)	U=1001	0.21
HFpEF	65 (65%)	26 (68%)	39 (63%)	χ²=0.16	0.65
NYHA functional classification				U=813	0.001
I	3 (3%)	1 (3%)	2 (3%)		
II	17 (17%)	11 (29%)	6 (10%)		
III	59 (59%)	23 (61%)	36 (58%)		
IV	21 (21%)	3 (8%)	18 (29%)		
Physical frailty	75 (75%)	34 (89%)	41 (66%)	χ²=5.7	0.009
Social frailty	82 (82%)	33 (87%)	49 (79%)	χ²=0.52	0.43
Cognitive frailty	38 (38%)	14 (37%)	24 (39%)	χ²=0	1.00
Walking start date after hospitalization, days	5 (3–7)	5 (3–8)	5 (2–7)	U=1010	0.23
Time of rehabilitation per day, min	40 (36–50)	41 (35–52)	40 (36–47)	U=1151	0.85
Rehabilitation program					
Gait training	99 (99%)	38 (100%)	61 (98%)	N/A	1
Muscle strength training	86 (86%)	32 (84%)	54 (87%)	χ²=0.01	0.92
Balance training	20 (20%)	6 (16%)	14 (23%)	χ²=0.32	0.57
Aerobic exercise	25 (25%)	7 (18%)	18 (29%)	χ²=0.91	0.34
Length of hospital stay, days	22 (16–28)	22 (19–30)	22 (14–27)	U=996	0.20

The non-improved group had a significantly higher prevalence of physical frailty (89% vs 66%, P=0.009) and showed a significant difference in the distribution of New York Heart Association (NYHA) functional classes (P=0.001) when compared with the improved group. However, no significant differences in age (86 years (IQR, 78-91) vs 82 years (IQR, 77-88)), sex (male; 34% vs 47%), walking initiation date (5 days (IQR, 3-8) vs 5 days (IQR, 2-7)), or rehabilitation program were observed between the groups.

Short physical performance battery

Table 2 presents the SPPB results at the initiation of cardiac rehabilitation and discharge. The initial SPPB score was seven or more points in 64% of patients, with no significant difference in the initial SPPB scores between the two groups. However, when compared with the non-improved group, the improved group showed significant increases in the total SPPB scores and all subitem scores (all P<0.001).

**Table 2:** Changes in the short physical performance battery scores from rehabilitation initiation to discharge Data are expressed as the median (interquartile range). Statistical comparisons were performed using the Mann–Whitney U test. SPPB, short physical performance battery

	All (N=100)	Non-improved group (n=38)	Improved group (n=62)	U-value	P-value
SPPB at rehabilitation initiation	6 (3–9)	5 (3–8)	6 (4–9)	1042	0.33
SPPB at discharge	7 (4–11)	4 (3–8)	10 (6–11)	492	<0.001
Change of SPPB	1 (0–2)	0 (-1–0)	2 (1–3)	0	<0.001
Change in SPPB subitems					
Walking speed	0 (0–1)	0 (0–0)	0 (0–1)	506	<0.001
Getting up from a chair	0 (0–1)	0 (0–0)	1 (0–1)	538	<0.001
Standing balance	0 (0–1)	0 (-1–0)	1 (0–1)	442	<0.001

Multivariate analysis

Table 3 shows the factors associated with improvement in physical performance, as identified by the modified Poisson regression analysis. Physical frailty (RR=0.64, 95% CI: 0.49-0.84) and the NYHA classification (RR=1.53, 95% CI: 1.21-1.93) were identified as factors significantly associated with improved physical performance.

**Table 3:** Modified Poisson regression analysis of factors associated with improvement in physical function CI, confidence interval; NYHA, New York Heart Association; RR, risk ratio

	RR	95% CI	z-value	p-value
Age	0.99	0.97–1.01	-1.15	0.35
Sex, male	1.30	0.97–1.73	1.49	0.07
NYHA classification	1.53	1.21–1.93	2.30	<0.001
Physical frailty	0.64	0.49–0.84	-2.88	0.002

## Discussion

In this study, we investigated the effects of acute-phase cardiac rehabilitation on physical performance in older patients with heart failure, focusing on the role of physical frailty. We found that 62% of patients experienced improvements in their physical performance. Multivariable analysis indicated that physical frailty may inhibit the enhancement of physical performance. As physical frailty can be reversed through personalized exercise and nutritional interventions [22], the early identification of frailty and implementation of appropriate intervention programs may contribute to the development of future rehabilitation strategies for patients with acute-phase heart failure.

The median age of participants in this study was similar to that in prior studies conducted in Japan (ranging from 81-83 years) and reflects the nation’s aging trends [4,21]. However, while a previous nationwide survey reported a physical frailty rate of 56%, our study found a higher prevalence of 75% [4]. This discrepancy may be due to the exclusion of participants with good physical performance (indicated by an SPPB score of 12), as our study specifically focused on evaluating the potential for improvement in physical performance through cardiac rehabilitation. Additionally, the female sex is a known risk factor for physical frailty [23], and the higher proportion of women in our study population (55% compared with 44% in a previous study [4]) may have contributed to the increased rate of frailty. As indicated in a prior study, a median SPPB score of 6 at the start of rehabilitation highlights the need for intervention, as scores below seven are associated with an elevated risk of death and rehospitalization [6].

Kitzman et al. reported that a cardiac rehabilitation program lasting from early hospitalization to three months post-discharge improved physical performance, even in patients with frailty [8]. In contrast, our study’s multivariate analysis indicated that physical frailty may inhibit improvement in physical performance during hospitalization. Frailty, characterized by a decline in physical performance and delayed recovery, often requires sustained intervention [24]. In our study, the approximate three-week duration of cardiac rehabilitation during hospitalization may have been insufficient to enhance physical performance in patients with physical frailty. When rehabilitating older patients, the content, volume, and frequency of interventions are essential factors in enhancing physical performance [25,26]. Although the median time of cardiac rehabilitation per day in this study was 46 min, which is slightly above the national average in Japan (38 min/day) [21], physical therapy was conducted only once daily, whereas multiple daily sessions have been shown to promote functional recovery [27,28]. Additionally, a multicomponent approach including aerobic, resistance, and balance training is recommended for patients with frailty [29]. Thus, optimizing acute-phase cardiac rehabilitation by refining training content and increasing session frequency is essential for patients with frailty. Moreover, benefits may be gained by strengthening post-discharge follow-up systems such as outpatient cardiac rehabilitation and long-term care-supported rehabilitation.

This study had some limitations; first, we were unable to evaluate physical performance prior to hospitalization, and differences in functional recovery might have depended on whether the initial performance decline was acute. Therefore, future studies should consider assessing physical performance prehospitalization. Second, the reliability of the questionnaire for patients with cognitive decline should be considered. In this study, we employed the questionnaire method recommended by previous research to assess physical and social frailty [16,30]. However, the reliability of the questionnaire may be compromised in cases of cognitive decline.

## Conclusions

Our study demonstrated that 62% of older patients with heart failure who underwent acute-phase cardiac rehabilitation achieved improved physical performance. Furthermore, we clarified that physical frailty acts as an inhibitory factor against improvement in physical performance, which is clinically meaningful and underscores the need for further investigation. These findings highlight the importance of routinely assessing physical frailty early during hospitalization and developing individualized rehabilitation plans based on this assessment. Furthermore, the results emphasize the necessity of establishing home-based rehabilitation programs following hospital discharge.
